# Psychosocial and behavioral problems of children and adolescents in the early stage of reopening schools after the COVID-19 pandemic: a national cross-sectional study in China

**DOI:** 10.1038/s41398-021-01462-z

**Published:** 2021-06-03

**Authors:** Lin Wang, Yiwen Zhang, Li Chen, Jianhong Wang, Feiyong Jia, Fei Li, Tanya E. Froehlich, Yan Hou, Yan Hao, Yuan Shi, Hongzhu Deng, Jie Zhang, Linjuan Huang, Xianghui Xie, Shuanfeng Fang, Liang Xu, Qi Xu, Hongyan Guan, Weijie Wang, Jianna Shen, Ying Qian, Xi Wang, Ling Shan, Chuanxue Tan, Yabin Yu, Xiaoyan Wang, Fangfang Chen, Lili Zhang, Xiaomeng Li, Xinmiao Shi, Xiaoyan Ke, Tingyu Li

**Affiliations:** 1 Department of Child Health Care, Children’s Hospital, Capital Institute of Pediatrics, Beijing, China; 2 Department of Developmental and Behavioral Pediatrics, Shanghai Children’s Medical Center Affiliated to Medical School of Shanghai Jiaotong University, Shanghai, China; 3 National Clinical Research Center for Child Health and Disorders, Ministry of Education Key Laboratory of Child Development and Disorders, Children’s Hospital of Chongqing Medical University, Chongqing, China; 4 Department of Developmental-Behavioral Pediatrics, the First Hospital of Jilin University, Changchun, Jilin, China; 5 Department of Developmental-Behavioral Pediatrics, Xinhua Hospital Affiliated to Medical School of Shanghai Jiaotong University, Shanghai, China; 6 Department of Pediatrics, University of Cincinnati College of Medicine and Cincinnati Children’s Hospital Medical Center, Cincinnati, OH USA; 7 Department of Biostatistics, Peking University, Beijing, China; 8 Department of Child Health Care,Tongji Hospital, Tongji Medical College, Huazhong University of Science and Technology, Wuhan, Hubei China; 9 Child Developmental & Behavioral Center, The Third Affiliated Hospital of Sun Yat-sen University, Guangzhou, China; 10 Department of Child Health Care, Xi’an Children’s Hospital, Xi’an, Shaanxi China; 11 Health Management Center, Fuzhou Children’s Hospital of Fujian Medical University, Fuzhou, China; 12 Children’s Hospital, Capital Institute of Pediatrics, Beijing, China; 13 Department of Child Health Care, Children’s Hospital Affiliated to Zhengzhou University, Zhengzhou, China; 14 Child Mental Health Research Center, Brain Hospital Affiliated to Nanjing Medical University, Nanjing, Jiangsu China; 15 Department of Early Childhood Development, Capital Institute of Pediatrics, Beijing, China; 16 Shanghai Pudong Institute of Education Development, Shanghai, China; 17 Chongqing educational science research academy, Chongqing, China; 18 Peking University Sixth Hospital, Peking University Institute of Mental Health, National Clinical Research Center for Mental Disorders, NHC Key Laboratory of Mental Health (Peking University), Beijing, China; 19 Department of Epidemiology, Capital Institute of Pediatrics, Beijing, China

**Keywords:** Psychiatric disorders, Scientific community, Human behaviour

## Abstract

This study aims to explore the psychosocial and behavioral problems of children and adolescents in the early stage of reopening schools. In this national cross-sectional study, a total of 11072 students from China were naturally divided into two groups based on their schooling status: reopened schools (RS) and home schooling (HS) group. The psychosocial and behavioral functioning were measured by Achenbach Child Behaviour Checklist (CBCL) and compared in these two groups. Multivariable logistic regression analyses were conducted to explore the independent predictors associated with the psychosocial and behavioral problems. Our results showed that the students in the RS group had more adverse behaviors than that of HS group. The RS group had the higher rates of parent-offspring conflict, prolonged homework time, increased sedentary time and sleep problems (all *p* < 0.001). When separate analyses were conducted in boys and girls, the RS group had the higher scores for (1) overall behavioral problems (*p* = 0.02 and *p* = 0.01), internalizing (*p* = 0.02 and *p* = 0.02) and externalizing (*p* = 0.02 and *p* = 0.004) behaviors in the 6–11 age group; (2) externalizing (*p* = 0.049 and *p* = 0.006) behaviors in the 12–16 age group. Multivariable regression showed parent-offspring conflict and increased sedentary time were the most common risk factors, while physical activity and number of close friends were protective factors for behavior problems in RS students (*p* < 0.01 or 0.05). The present study revealed that students’ psychosocial and behavioral problems increased in the early stage of schools reopened unexpectedly. These findings suggest that close attention must be paid and holistic strategies employed in the school reopening process of post-COVID-19 period.

## Introduction

In the past year, the world saw the coronavirus disease (COVID-19) outbreak affect countries in waves more widespread on a global scale than SARS and other epidemics^1–3^. According to the official website of the World Health Organization, more than 17,000,000 people have been confirmed to have COVID-19 globally as of July 31, 2020^4^. To better fight against the epidemic, social distancing measures have been implemented in many countries to ease the burden on health systems. Most governments around the world have temporarily closed educational institutions in an attempt to contain the spread of the COVID-19 pandemic, thereby impacting over 60% of the world’s student population^5^.

A nationwide closure of educational institutions was first implemented as an emergency measure in China in February 2020. In order to mitigate the negative consequences on students during home confinement, the government, National Health Commission, medical health specialists, schools and parents worked together to provide activities to maintain routines and distract children from the harsh reality of the epidemic^6–9^. Meanwhile, online services to help the public cope with mental health issues were implemented in a large number of cities^7,10,11^. Measures undertaken to further minimize adverse social-emotional effects of school closures included increased offerings for parent-offspring activities, a reduction in academic load, and a shift in the routine communication of daily life from the schools to the online class clusters in home schooling programs^12,13^.

Many studies have shown the adverse aspects of school closure and assumed that resumption of in-person schooling would end these negative impacts on the psychosocial well-being of children and adolescents^14,15^. Despite attention to the mental health impact of school closures and stay at home orders, no research has described psychosocial and behavioral effects of returning to schools after prolonged home confinement and online schooling.

Since COVID-19 was contained in China in late spring to summer 2020, the schools have been entering the reopening phase since April 2020 following the principle of “No new COVID-19 cases diagnosed over the previous 21 days in communities free of the disease” from the national and local Centres for Disease Control and Prevention based on the incubation period of this virus^16–18^. As the timetable of schools reopening varies according to the guidelines set by each city, we have had the rare opportunity to observe the psychosocial and behavioral problems of children and adolescents in the early period of reopening schools after the COVID-19 pandemic compared to that of continued home schooling.

## Methods

### Design, participants and procedure

This is a national cross-sectional study of Chinese students from primary, junior and high schools performed via an online survey running from May 20 to June 13, 2020. This survey period corresponds to the end stage of school closure and the reopening of schools throughout the country after the containment of COVID-19 in China.

**Fig. 1:**
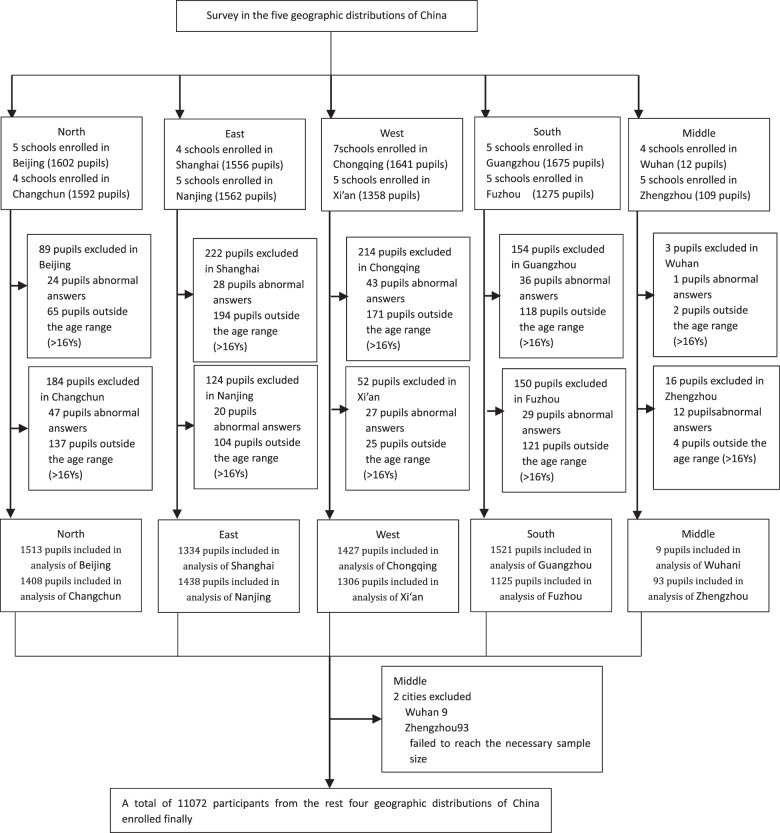
Flow diagram illustrating the survey profile. The study population was selected according to geographical regions of China.

The study was prospectively sponsored by the Subspecialty Group of Developmental and Behavioral Paediatrics, the Society of Paediatrics, the Chinese Medical Association on April, when the COVID-19 epidemic in China was nearly controlled and cities were ready to resume typical activities after a long-term lockdown. The study population was selected according to geographical regions (North, East, West, South and Middle) of China. The capital city with the largest population and a capital city geographically in the center of the region were selected to form a representative sample of the population. Therefore, 10 cities in the North (Beijing, Changchun), East (Shanghai, Nanjing), West (Chongqing, Xi’an), South (Guangzhou, Fuzhou) and Middle (Wuhan, Zhengzhou) regions were selected. Within each city, the district with the median income was selected. One primary, one junior and one high school were selected resulting in a pool of eligible regular public schools that were of a medium-size based on public information, contained at least 1000 pupils, had no more than 60% of pupils of the same sex and were active for more than ten years in the urban and rural areas of the district. In order to reach the necessary sample size, two classes were randomly selected from each grade level of the urban and rural schools. The participants from the ten cities in five geographic regions of China were divided into two groups according to their schooling status: the home schooling group (HS group) and the reopened school group (RS group), for which schools had reopened for at least 2 weeks and no more than 2 months. The study profile was described in Fig. 1.

This study was conducted with IRB approval at Capital Institute of Pediatrics (Number: SHERLL2020019). All participants provided their online informed consent.

### Questionnaire and measures

Because it was not feasible to conduct face-to-face surveys during this special period, the data was collected by an online survey using the Wenjuanxing platform (https://dnalab.wjx.cn/jq/75239406.aspx). The parent report questionnaire was sent to the recruited parents of the students by their educational institutions. The questionnaire covered three thematic areas:

#### Demographics

The demographic variables included age, sex, residential place (urban or rural), maternal education status (≤9 years or >9 years), parents having organic diseases (yes or no) and family income (reduced orno change), et al.

#### Psychosocial impacts

The psychosocial variables included parent-offspring conflict (Yes/No), homework time (≤2 h, >2 h per day), sedentary time (≤6 h, >6 h per day), screen exposure time (≤4 h, >4 h per day), sleep problems (Yes/No), physical activity (≤1 h, >1 h per day) and number of close friends (≥4 or <4).

#### Emotional and behavioral problems

The Parent Achenbach Child Behaviour Checklist (CBCL), a widely used, empirically derived measure to assess the dimensional psychopathology and adaptive functioning in children, which has a high test–retest stability and good internal consistency was administered^19^. The 113-item scale uses a 3-point Likert scale (not true, somewhat or sometimes true and very often or always true) and is given to parents to measure a wide range of child behaviors across the past six months. The Chinese version of CBCL contains ten empirically-based syndrome scales related to psychiatric problems: anxious/depressed, withdrawn/depressed, somatic complaints, social problems, thought problems, attention problems, rule-breaking behavior, aggressive behavior, an internalizing and externalizing broad band score and a total score^20^. The scoring system is sex-based and has different cut-off points for clinical significance for the various age groups. The presence of a behavioral disorder is indicated when a participant’s score exceeds the threshold for clinical significance on any of the subscales.

### Quality control

The same electronic device could be used only once to complete the questionnaire, which did not collect any personal information such as names, thereby ensuring anonymity and honest responses. When designing the online questionnaire, we added items that could not be submitted if there were missing items to improve the effectiveness of the questionnaire. The questionnaire settings (e.g., required questions and limiting the scope of questions) provided control over the questionnaire and prevented respondents from randomly selecting responses or trying to complete the survey as quickly as possible.

### Statistical analyses

The mean and standard deviation (SD) for normal continuous variables and the frequency and percentage per category for categorical variables were used to analyse the demographic and psychosocial characteristics of students in each group (the HS group vs. RS group). Group differences were compared using independent *t*-tests for continuous variables and chi-square tests for categorical variables.

Cronbach’s α was employed to evaluate the internal consistency of the total and subscale CBCL scores. We further compared the total and subscale scores between the two groups using the General Linear Model (GLM) Analysis of Co-variance (ANCOVA) for boys and girls aged 6–11 and 12–16 respectively, with age as a covariate.

To identify independent predictors contributing to the presence of behavioral disorder that total score of overall behavioral problems exceeds the cut-off points in the various age and sex different subgroupings, multivariate logistic regression analysis was performed using a stepwise variable selection procedure in RS and HS group respectively. Odds ratio (OR) and 95% CI were evaluated to assess associations. Statistical significance was defined as a two-sided *p*-value less than 0.05. All analyses were performed using SAS 9.4 (SAS Institute, Cary, NC).

## Results

Nationwide, a total of 12,382 participants from five geographic regions of China were enrolled in the survey. The cities of Wuhan and Zhengzhou were excluded from the survey because they did not reach the necessary sampling size (only 12 for Wuhan, 109 for Zhengzhou). Of the remaining 12,261 participants from 40 schools (8 were junior and senior combined high schools), a total of 11,072 (90.3%) valid questionnaires were obtained after removing 254 responses with suspect answers (contradictions and/or inconsistencies) and 935 responses that were outside the age range (>16 years) (Fig. 1). The Cronbach’s α were above 0.7 for all subscales (except the sexual problem in age 6–11 is 0.571) for both sexes in our sample, indicating acceptable internal consistency.

**Fig. 2:**
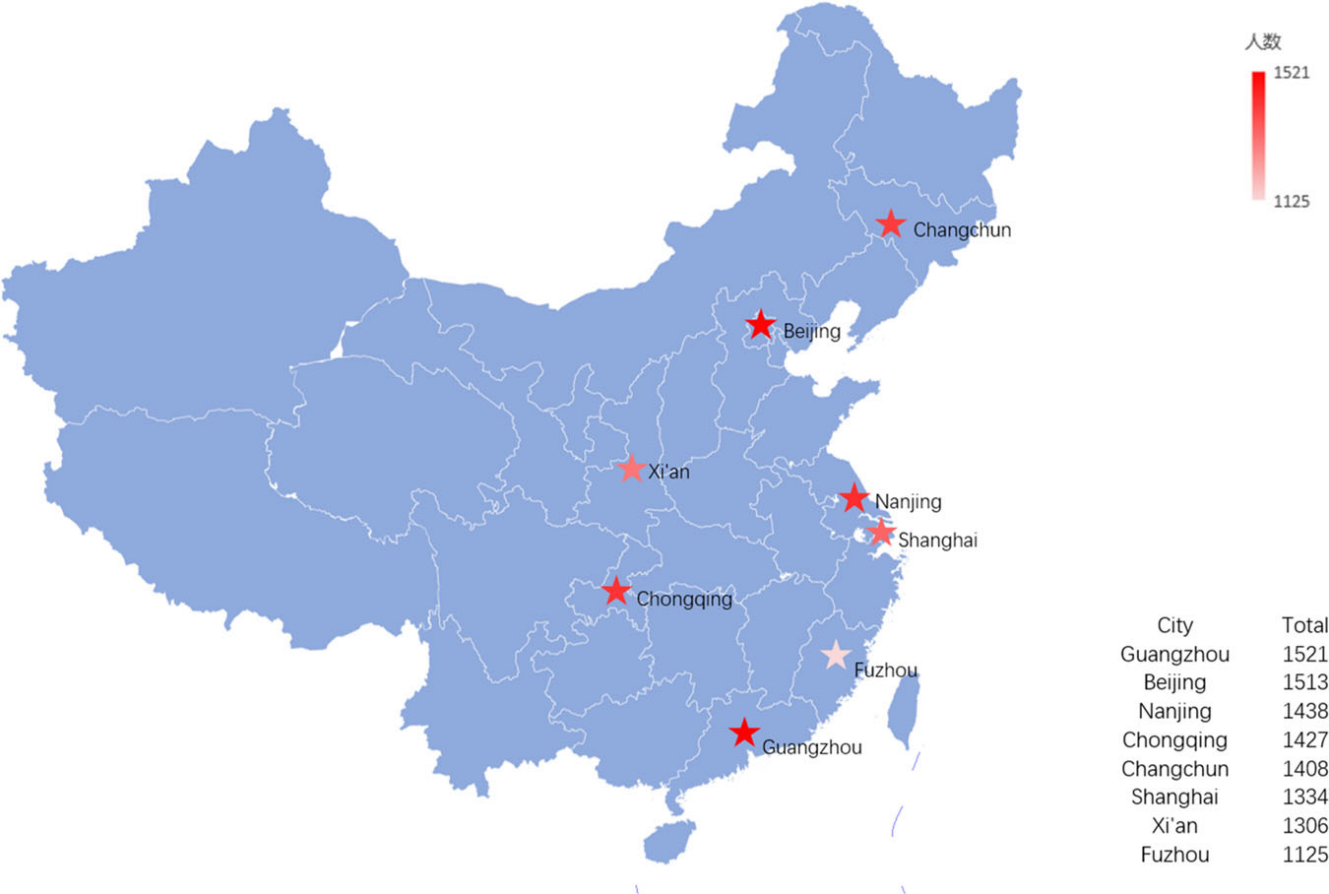
Sampling distributions. The 8 cities in the North (Beijing, Changchun), East (Shanghai, Nanjing), West (Chongqing, Xi’an) and South (Guangzhou, Fuzhou) regions of China finally enrolled and the valid number in each city.

Figure 2 displays data from the eight capital and municipal cities eventually enrolled from the four geographic regions.

Table 1 presents the sociodemographic features of the whole sample and compares the 7453 students in the RS group to the 3619 students in the HS group.

**Table 1:** Demographic and psychosocial characteristic of RS group and HS group during COVID-19.

Characteristics	RS group (*n* = 7453)	HS group (*n* = 3619)	Total (*n* = 11072)	Z/chi-square	*P*-value
Age (years)					
6–11	9.2 (1.32)	9.0 (1.34)	9.1 (1.33)	−6.00	<0.001
12–16	13.9 (1.38)	13.9 (1.44)	13.9 (1.40)	−1.02	0.31
Sex				1.38	0.24
Male	3911 (52.5%)	1856 (51.3%)	5767 (52.1%)		
Female	3542 (47.5%)	1763 (48.7%)	5305 (47.9%)		
Residential place				0.26	0.61
Urban	4260 (57.2%)	2050 (56.6%)	6310 (57.0%)		
Rural	3193 (42.8%)	1569 (43.4%)	4762 (43.0%)		
Maternal education status				126.96	<0.001
≤9 years	4345 (58.3%)	2511 (69.4%)	6856 (61.9%)		
>9years	3108 (41.7%)	1108 (30.6%)	4216 (38.1%)		
Parents having organic diseases				17.31	<0.001
Yes	147 (2.0%)	118 (3.3%)	265 (2.4%)		
No	7306 (98.0%)	3501 (96.7%)	10807 (97.6%)		
Family income				0.44	0.505
Reduced	3671 (49.3%)	1807 (49.9%)	5478 (49.5%)		
No change/ Increased	3782 (50.7%)	1812 (50.1%)	5594 (50.5%)		
Parent-offspring conflict				20.58	<0.001
No	2391 (32.1%)	1318 (36.4%)	3709 (33.5%)		
Yes	5062 (67.9%)	2301 (63.6%)	7363 (66.5%)		
Sedentary Time (hours)				36.55	<0.001
≤6	5163 (69.3%)	2708 (74.8%)	7871 (71.1%)		
>6	2290 (30.7%)	911 (25.2%)	3201 (28.9%)		
Homework time (hours)				76.93	<0.001
≤2	4114 (55.2%)	2315 (64.0%)	6429 (58.1%)		
>2	3339 (44.8%)	1304 (36.0%)	4643 (41.9%)		
Screen exposure time (hours)				1.07	0.30
≤4	4320 (58.0%)	2135 (59.0%)	6455 (58.3%)		
>4	3133 (42.0%)	1484 (41.0%)	4617 (41.7%)		
Physical activity time(hours)				279.85	<0.001
≤1	4338 (58.2%)	1494 (41.3%)	5832 (52.7%)		
>1	3115 (41.8%)	2125 (58.7%)	5240 (47.3%)		
Sleep problems				13.13	<0.001
No	5158 (69.2%)	2626 (72.6%)	7784 (70.3%)		
Yes	2295 (30.8%)	993 (27.4%)	3288 (29.7%)		
Number of close friends				0.89	0.344
<4	4281 (57.4%)	2113 (58.4%)	6394 (57.7%)		
≥4	3172 (42.6%)	1506 (41.6%)	4678 (42.3%)		

COVID-19 vs. coronavirus disease 2019; *RS group* reopened school group, *HS groups* home schooling group

Data are mean (SD) or *n* (%). Effect size is estimated by ^*^Cohen’s d or ^#^phi coefficient.

### Psychosocial features of RS and HS group

The RS group showed a higher prevalence rate for parent-offspring conflict (67.9 vs. 63.6%, *p* < 0.001), prolonged homework time (>2 h per day) (44.8 vs. 36.0%, *p* < 0.001), increased sedentary time (>6 hours per day) (30.7 vs. 25.2%, *p* < 0.001), sleep problems (30.8 vs. 27.4%, *p* < *0*.001), as well as physical inactivity time (≤1 hour per day) (58.2 vs. 41.3%, *p* < 0.001) than the HS group, as described in Table 1.

### Behavioral characteristics by group for students age 6–11

The RS group had a significantly higher CBCL overall score for problem behaviors compared to that of the HS group for both sexes (16.79 vs. 14.87, *p* = 0.02 and 13.61 vs. 11.62, *p* = 0.01 respectively) (Table 2). Both the internalizing and externalizing behavior scores of the RS group were higher than the HS groupin boys (8.34 vs. 7.14, *p* = 0.02 and 8.79 vs. 7.87, *p* = 0.02, respectively) and in girls (5.58 vs. 4.73, *p* = 0.02 and 7.17 vs. 6.07, *p* = 0.004 respectively). The difference in each subscale for both sexes between the RS group and HS group were detailed in Table 2.

**Table 2:** Comparison of CBCL scores between RS group and HS group (boys and girls of 6–11 years) during COVID-19.

Behavior subscales	RS group	HS group	*F*	*P-*value	Cronbach’s Alpha
**Boys**	***n*** = **1747**	***n*** = **1070**			
Schizoid	1.41 (0.05)	1.33 (0.06)	0.98	0.32	0.744
Depression	1.98 (0.09)	1.61 (0.11)	6.91	0.009	0.896
Social problems	1.22 (0.05)	0.95 (0.06)	13.95	<0.001	0.775
Compulsive activity	2.18 (0.08)	1.83 (0.10)	6.82	0.01	0.866
Somatic complaints	0.60 (0.04)	0.58 (0.05)	0.04	0.85	0.851
Social withdrawal	0.95 (0.04)	0.83 (0.05)	3.30	0.07	0.769
Hyperactivity	3.22 (0.08)	2.85 (0.10)	8.38	0.004	0.826
Aggressive behavior	4.51 (0.13)	4.14 (0.17)	2.98	0.08	0.906
Delinquent behavior	1.06 (0.05)	0.88 (0.07)	4.70	0.03	0.841
Internalizing behavior	8.34 (0.31)	7.14 (0.40)	5.64	0.02	0.930
Externalizing behavior	8.79 (0.24)	7.87 (0.31)	5.47	0.02	0.835
Total score	16.79 (0.52)	14.87 (0.66)	5.20	0.02	0.974
**Girls**	***n*** = **1445**	***n*** = **986**			
Depression	2.41 (0.10)	2.09 (0.12)	4.62	0.03	0.874
Social withdrawal	1.39 (0.06)	1.20 (0.08)	3.66	0.06	0.831
Somatic complaints	0.99 (0.06)	0.85 (0.07)	2.47	0.12	0.832
Schizoid/Compulsive activity	0.78 (0.05)	0.59 (0.06)	7.30	0.007	0.810
Hyperactivity	2.83 (0.09)	2.45 (0.11)	7.51	0.006	0.836
Sexual problem	0.63 (0.03)	0.56 (0.03)	2.53	0.11	0.571
Delinquent behavior	0.28 (0.02)	0.21 (0.03)	3.77	0.05	0.748
Aggressive behavior	3.75 (0.13)	3.15 (0.15)	8.96	0.003	0.896
Cruel	0.31 (0.03)	0.27 (0.03)	1.19	0.28	0.795
Internalizing behavior	5.58 (0.24)	4.73 (0.29)	5.17	0.02	0.897
Externalizing behavior	7.17 (0.24)	6.07 (0.29)	8.20	0.004	0.767
Total score	13.61 (0.50)	11.62 (0.60)	6.44	0.01	0.972

COVID-19 vs. coronavirus disease 2019; *RS group* reopened school group, *HS group* home schooling group

General Linear Model (GLM) Analysis of Co-variance (ANCOVA) were employed to compare the total and subscale scores between the two groups, with age as covariate. Scores of the two groups are Least Squares Means (SE). Cronbach’s Alpha were generated from Pearson Correlation to evaluate the internal consistency of the total and subscale scores.

### Behavioral characteristics by group for students age 12–16

The RS group had a significantly higher CBCL score than the HS group for the two externalizing behaviors for both sexes (Table 3), which resulted in a significantly higher externalizing score for the RS group than the HS group in both boys (9.42 vs. 8.35, *p* = 0.049) and girls (6.20 vs. 5.11, *p* = 0.006).

**Table 3:** Comparison of CBCL scores between RS group and HS group (boys and girls of 12–16 years) during COVID-19.

Behavior subscales	RS group	HS group	*F*	*P-*value	Cronbach’s Alpha
**Boys**	***n*** = **2164**	***n*** = **786**			
Somatic complaints	1.12 (0.06)	1.16 (0.10)	0.10	0.76	0.892
Schizoid	0.97 (0.04)	1.08 (0.07)	1.91	0.17	0.79
Social problems	2.17 (0.08)	2.02 (0.14)	0.88	0.35	0.902
Immature	0.98 (0.03)	0.91 (0.06)	1.09	0.30	0.713
Compulsive activity	1.15 (0.04)	1.14 (0.07)	0.00	0.95	0.782
Hostility	1.68 (0.07)	1.68 (0.11)	0.00	0.99	0.869
Delinquent behavior	1.37 (0.05)	1.18 (0.09)	3.39	0.07	0.834
Aggressive behavior	3.57 (0.11)	3.01 (0.19)	6.56	0.01	0.916
Hyperactivity	2.80 (0.07)	2.48 (0.11)	5.71	0.02	0.824
Internalizing behavior	6.39 (0.24)	6.31 (0.40)	0.03	0.87	0.907
Externalizing behavior	9.42 (0.28)	8.35 (0.46)	3.88	0.049	0.911
Total score	14.18 (0.47)	13.41 (0.78)	0.71	0.40	0.976
**Girls**	***n*** = **2097**	***n*** = **777**			
Anxiety/Compulsive activity	2.72 (0.11)	2.57 (0.18)	0.50	0.48	0.921
Somatic complaints	0.70 (0.04)	0.69 (0.06)	0.00	0.95	0.832
Schizoid	0.78 (0.04)	0.78 (0.07)	0.00	0.99	0.839
Depression/withdrawal	1.91 (0.08)	1.79 (0.13)	0.65	0.42	0.893
Immature	2.48 (0.07)	2.33 (0.12)	1.11	0.29	0.837
Delinquent behavior	2.41 (0.07)	2.01 (0.12)	7.44	0.006	0.854
Aggressive behavior	3.20 (0.11)	2.57 (0.18)	9.33	0.002	0.912
Cruel	0.60 (0.04)	0.52 (0.06)	1.02	0.31	0.833
Internalizing behavior	6.10 (0.24)	5.84 (0.40)	0.33	0.57	0.874
Externalizing behavior	6.20 (0.20)	5.11 (0.34)	7.69	0.006	0.842
Total score	13.69 (0.48)	12.36 (0.78)	2.09	0.15	0.977

COVID-19vs. coronavirus disease 2019; *RS group* reopened school group, *HS group* home schooling group

General Linear Model (GLM) Analysis of Co-variance (ANCOVA) were employed to compare the total and subscale scores between the two groups, with age as covariate. Scores of the two groups are Least Squares Means (SE). Cronbach’s Alpha were generated from Pearson Correlation to evaluate the internal consistency of the total and subscale scores.

### Risk factors of psychosocial and behavioral problems in RS and HS group

Table 4 showed the independent variables which were significantly associated with behavioral problems (as measured by total score of overall behavioral problems across sex and age subgroups) in RS and HS group respectively. The parent-offspring conflict and increased sedentary time were the most common risk factors, while physical activity and number of close friends were protective factors in RS group (*p* < 0.01 or 0.05). In the HS group, physical inactivity and screen exposure time were risk factors (*p* < 0.01 or 0.05).

**Table 4:** Multivariate logistic regression analyses of psychosocial and behavioral problems and impact factors in different age and sex subgroups.

Variables	β	OR (95% CI)	*P-*value
Boys of 6–11			
RS group			
Physical activity (>1 h vs. ≤1 h per day)	−0.4268	0.653 (0.454 to 0.937)	0.021
Number of close friends (≥4 vs. <4)	−0.4683	0.626 (0.431 to 0.909)	0.014
Screen exposure time (>4 h vs. ≤4 h per day)	0.6890	1.992 (1.403 to 2.828)	<0.001
HS group			
Homework time (>2 h vs. ≤2 h per day)	0.5655	1.760 (1.101 to 2.814)	0.018
Parent-offspring conflict (Yes vs. No)	0.8443	2.326 (1.234 to 4.384)	0.009
Number of close friends (≥4 vs. <4)	−0.7775	0.460 (0.260 to 0.811)	0.007
Girls of 6–11			
RS group			
Family income (Reduced vs. No change)	0.6653	1.945 (1.238 to 3.056)	0.004
Parent-offspring conflict (Yes vs. No)	1.3089	3.702 (1.819 to 7.533)	<0.001
Physical activity (>1 h vs. ≤1 h per day)	−0.4758	0.621 (0.390 to 0.990)	0.045
Screen exposure time (>4 h vs. ≤4 h per day)	0.6486	1.913 (1.226 to 2.985)	0.004
HS group			
Family income (Reduced vs. No change)	0.7848	2.192 (1.138 to 4.221)	0.019
Screen exposure time (>4 h vs. ≤4 h per day)	1.0070	2.738 (1.473 to 5.087)	0.001
Boys of 12–16			
RS group			
Age (years)	−0.1808	0.835 (0.748 to 0.932)	0.001
Family income (Reduced vs. No change)	0.4295	1.536 (1.139 to 2.073)	0.005
Sedentary time (>6 h vs. ≤6 h per day)	0.5627	1.755 (1.306 to 2.359)	<0.001
Parent-offspring conflict (Yes vs. No)	0.6512	1.918 (1.311 to 2.805)	<0.001
Parents having organic diseases (Yes vs. No)	1.4493	4.260 (2.247 to 8.076)	<0.001
Number of close friends (≥4 vs. <4)	−0.5804	0.560 (0.409 to 0.766)	<0.001
HS group			
Homework time (>2 h vs. ≤2 h per day)	−0.7274	0.483 (0.267 to 0.875)	0.016
Physical activity (>1 h vs. ≤1 h per day)	−0.7803	0.458 (0.267 to 0.788)	0.005
Girls of 12–16			
RS group			
Maternal education status (>9 years vs. ≤9 years)	−0.5311	0.588 (0.413 to 0.837)	0.003
Sedentary time (>6 h vs. ≤6 h per day)	0.3691	1.446 (1.030 to 2.032)	0.033
Screen exposure time (>4 h vs. ≤4 h per day)	0.4186	1.520 (1.057 to 2.184)	0.024
Parent-offspring conflict (Yes vs. No)	0.4611	1.586 (1.086 to 2.316)	0.017
Physical activity (≤1 h vs. >1 h per day)	−0.4367	0.646 (0.448 to 0.932)	0.020
Number of close friends (≥4 vs. <4)	−0.6610	0.516 (0.363 to 0.734)	<0.001
HS group			
Parents having organic diseases (Yes vs. No)	0.9493	2.584 (1.138 to 5.865)	0.023
Physical activity (>1 h vs. ≤1 h per day)	−0.8195	0.441 (0.258 to 0.753)	0.003

*RS group* reopened school group, *HS group* home schooling group, *OR* odds ratio, *CI* confidence interval.

Multivariate logistic regression analysis was performed using stepwise variable selection procedure to identify independent predictors contributing to the presence of behavioral disorder that total score exceeds the cut-off point in different group. In RS and HS group, variables inserted into the model were age, residential place, maternal education status, parents having organic diseases, family income, parent-offspring conflict, homework time, sedentary time, screen exposure time physical activity and number of close friends.

## Discussion

This study reports the overall psychosocial and behavioral impact on children and adolescents of long-term home confinement and the early stage of reopening schools during the COVID-19 pandemic in China. The reopening of schools at different times set by local education departments across the country provided an opportunity to evaluate the behavioral impact on children and adolescents over the naturally occurring course of reopening schools versus continued home schooling. To our knowledge, this study is the first national cross-sectional survey to explore the psychosocial impact of reopening schools after long-term home confinement and online schooling, including the identification of risk and protective factors during these two phases. Our findings highlight the need for vigilance regarding the psychological needs of children and adolescents after as well as during epidemics, and may provide key knowledge needed to formulate post-COVID-19 recovery strategies.

Compared with the HS group, the RS group showed higher rates of parent-offspring conflict, prolonged homework time, increased sedentary time, sleep problems, as well as physical inactivity. Moreover, the RS group displayed higher emotional and behavioral problem scores as well as positive detection rate (Supplementary Table 1) than those of HS group unexpectedly. The scores of the RS group were higher than those of the HS group in both internalizing and externalizing behavior problems for both sexes in the children aged 6–11 years and for two externalizing behavior subscales for both sexes in adolescents aged 12–16 years. Specifically, children aged 6–11 who returned to school showed more depression, compulsive behavior and hyperactivity, while adolescents of age 12–16 showed more aggressive behavior, compared to those who were home schooled. Of note, our finding of increased social-emotional problems for children in the RS group compared to the HS group is consistent with those of a recent study of adults, which showed increased psychological problems for medical imaging workers during the late/reopening stage of the epidemic in China^21^. Our study also identified risk and protective factors for behavior issues in children and adolescents whose schools reopened. Multivariable regression showed that parent-offspring conflict, increased screen exposure time and sedentary time were linked to an increased odds of the CBCL total behavioral score exceeding the threshold for clinical significance in the RS group, while physical activity and number of close friends were the most common protective factors among RS students.

As COVID-19 is much more widespread than other epidemics and has affected countries in waves, the impact of school closures across the world has been more extensive and felt more profoundly than in other recent infectious disease outbreaks^22–24^. Previous studies have demonstrated that in addition to the increase in clinging, inattentive and irritable documented at the beginning of the epidemic, with its link to disrupted school and daily routine, poor dietary habits (obesity), increased use of electronic devices, can further aggravate adverse effects on children and adolescents^25–27^. School reopening was assumed to be the most effective measure for alleviating the negative effects of home quarantining and improving the psychosocial well-being of children^28,29^. However, contrary to our expectation, our study showed that the psychosocial behavioral problems in the early stage of school reopening were still present and, in fact, students in the RS group exhibited more psychological problems across most CBCL subscales than that of the HS group.There may be the potential explanations for this phenomenon. First, due to concerns regarding an impending psychological crisis for children during home confinement, the Chinese government, National Health Commission, medical health specialists and schools took steps to reduce academic pressure of online home school courses (compared to the academic load for in-person school) and implemented the psychological interventions for young children^8,12,13^. Since it was expected that children would easily adapt to the in-person school environment after long-term home schooling, special measures to ease the transition were not fully executed. However, when transitioning back to in-person school, children may react negatively to the re-imposition of rapid increase in academic pressure from parents and teachers, and may have more peer relationship problems, as well as difficulties adjusting to the changed daily school schedule. The reduced academic load during home schooling likely necessitated increased study once schools reopened to make up for the lost months and allow them to complete entire semester courses before mid-July, potentially resulting in excessive homework and restrictions on extra-curricular recreational activities. Previous studies have demonstrated that academic pressure was the most commonly identified stressor across students, irrespective of age and sex, which was largely driven by parental and teacher expectations^30,31^. Indeed, during this special period of school reopening, the sudden shift to strict, organized in-person schooling and the stark discrepancy between home and school environments may have created new psychological stressors for families, as demonstrated by the increase in parent-child conflict documented in the RS group. An additional explanation for the higher behavior problems scores observed in RS versus the HS group may be that some externalizing symptoms documented by the CBCL subscale (e.g., aggressive behavior, rule-breaking behavior) cannot be exhibited in the home circumstance if there is no opportunity for social interaction^32^.

As this national cross-sectional survey with a large sample size, some of effect sizes in our study were small but nevertheless significant (Supplementary Table 2), whereas in small sample they cannot be detectable. The differences in these factors between RS and HS groups might indicate that some psychological behaviors need further attention after the COVID-19 pandemic. Of note, these factors may be the early signs of depression or other mental health issues, or they could represent related phenotypes. Students with these factors need to be followed-up carefully and assessed when necessary in case the disease is at play.

From the estimation of effect sizes, we found that physical inactivity, increased sedentary time, and parent-offspring conflict contributed more in the psychopathology. The same results were also found from the multivariate logistic regression analyses on the presence of at least a positive screened dimension of internalizing and externalizing behavior in different age and sex subgroups (Supplementary Tables 3 and 4). We observed 58.1% of physical inactivity among children and adolescents in the RS group. An observational study among adolescents from countries in Europe and Latin America presented the similar high prevalence of not activity, 45.9% in 10–15 year-old and 54.1% in 16–19 year-old groups respectively^33^. It is well-established that physical inactivity leads to the development of sedentary behaviors with a subsequent negative impact on the physical, mental, and social health of children and adolescents^34,35^. Regular physical activities are vital to keep students’ fitness, and also may help students recuperate from the stress and anxiety from the quarantine during the COVID-19 crisis. Therefore, as schools begin to reopen, there is a need in terms of public health to ensure that students are effectively freed from restrictions on physical activity through progressive participation in physical activity^36,37^, eventually meeting the minimum recommendations for physical activity in the WHO guidelines^38^.

In this study, 67.9% guardians reported the parent-offspring conflict in the RS group. COVID-19 is a time of hardship for all family members. Parents or guardians may experience low income, unemployment, working remotely or being unable to work due to look after children, with no clarity on how long the situation will last^14^. Another reason leading to increased confliction is due to cultural context: academic performance is generally considered as the main metric for evaluating a student’s study efforts. Therefore, students who had underperformance at home found themselves in an even more difficult situation when returned to school with suddenly increased burden of structured lessons, hence schools and parents should appropriately adjust academic expectations in early stage of school returning and give students a reasonable transition period for school life.

Compared to previous studies, this study first identified the protective effect of the number of close friends. More close friends and peer support in the reopened school life may improve their psychosocial and behavioral well-being. Agarwal B and Brooks SK have reported peer support played an important role on the sustaining resilience in managing occupational stress^39^. In the early stage of reopening, the school should set up more group physical activities, fruitful peer support and communication to address their fears and concerns, and playing cooperative games to reduce loneliness^6^, which may be useful to support psychological resilience and relief externalizing or internalizing behaviors from the pandemic and home-confinement.

Therefore, teachers, parents and administrators should take into account that the adjustment difficulties students may have, and accordingly make some necessary adjustments of the academic load and physical activities at the early stage of schools reopening. They also should provide students with more peer support in communicating with friends/classmates, as well as reduce conflict with parents arising from their academic expectations. A rapid response support team (composed of teachers, professional counselors and school doctors) should be established to provide psychological support and help the students in the adjustment of sudden transfer from loose home-schooling to structured educational curriculum of reopening school. Students, who with pre-existing mental health illness, behavioral problems and developmental disorders, would need extra support after returning to school^6^. Regular psychological and behavioral screening of students should be carried out timely to find out the problems. Then, early intervention should be administrated timely and promptly by developmental behavior doctors, psychologists and psychiatrists. As students across the world are still facing the school reopening, pre-setting plan and coping strategies targeting these risk factors are required to address the unique needs at the early stage of school reopening.

The present study has limitations. First, a cross-sectional design was utilized while a longitudinal approach might help to determine whether the psychosocial behavioral problems and disorders identified in this study improve as students acclimatize to in-person schooling. Second, the survey was not executed by random sampling and was based on an online survey, making the extent to which this sample is nationally representative is uncertain. Third, the two groups were differentially distributed across cities, which may have influenced the results, although previous reports have indicated there were no significant differences in the psychosocial problems of children and adolescents among different large cities in China^40^. Fourth, the effect size was small in several variables as statistical power allows to detect even small effects in the large sample size, so the explanation of the results should be cautious.

In summary, we found that the psychological and behavioral symptoms documented among children and adolescents during the home school phase of the COVID-19 pandemic did not decrease as expected in the early stage of school reopening. This unexpected phenomenon observed at a unique time in human history will help us better understand the most important psychological needs of children and adolescents. These findings suggest that the mental health vulnerability does not spontaneously resolve with virus control. Rather, the early phase of school reopening remains an extremely challenging period for children and adolescents, requiring attention and collaboration from schools, families, mental health providers and policy-makers to protect the mental health of children and adolescents in the post-COVID-19 period. Since physical health, mental health, and productivity in adult life are deeply rooted in childhood psychosocial experience and environmental exposures^28^, more research is also necessary to incorporate the voices of children and their families when developing holistic strategies to prevent long-term consequences of the COVID-19 pandemic for the world’s children^41^.

## Supplementary information

Supplementary Tables
