# Supplementary material for: Psychosocial and behavioral problems of children and adolescents in the early stage of reopening schools after the COVID-19 pandemic: a national cross-sectional study in China
Source: Transl Psychiatry. 2021 Jun 3;11:342. doi: 10.1038/s41398-021-01462-z (PMC8172553; doi:10.1038/s41398-021-01462-z)
Supplement: Supplementary file 1 — Supplementary Tables [file 41398_2021_1462_MOESM1_ESM.docx]

**Supplementary Table 1. The Percentage of Children Who Were Screened Positive and comparison between RS Group and HS Group**

| **Behavior Subscales** **Positive** | **RS group** | **HS group** | **Total** | **Chi-square** | **P Value** |
| --- | --- | --- | --- | --- | --- |
| **Boys** **of 6-11ys** | **N=1747** | **N=1070** | **N=2817** |  |  |
| Schizoid | 65 (3.7%) | 39 (3.6%) | 104 (3.7%) | 0.01 | 0.918 |
| Depression | 68 (3.9%) | 30 (2.8%) | 98 (3.5%) | 2.34 | 0.126 |
| Social problems | 73 (4.2%) | 26 (2.4%) | 99 (3.5%) | 5.98 | 0.014 |
| Compulsive activity | 88 (5.0%) | 34 (3.2%) | 122 (4.3%) | 5.54 | 0.019 |
| Somatic complaints | 23 (1.3%) | 16 (1.5%) | 39 (1.4%) | 0.16 | 0.693 |
| Social withdrawal | 55 (3.1%) | 18 (1.7%) | 73 (2.6%) | 5.65 | 0.017 |
| Hyperactivity | 76 (4.4%) | 34 (3.2%) | 110 (3.9%) | 2.43 | 0.119 |
| Aggressive behavior | 46 (2.6%) | 22 (2.1%) | 68 (2.4%) | 0.94 | 0.333 |
| Delinquent behavior | 30 (1.7%) | 16 (1.5%) | 46 (1.6%) | 0.20 | 0.652 |
| Total score | 144 (8.2%) | 79 (7.4%) | 223 (7.9%) | 0.67 | 0.412 |
| **Girls** **of 6-11ys** | **N=1445** | **N=986** | **N=2431** |  |  |
| Depression | 25 (1.7%) | 13 (1.3%) | 38 (1.6%) | 0.65 | 0.422 |
| Social withdrawal | 30 (2.1%) | 22 (2.2%) | 52 (2.1%) | 0.07 | 0.795 |
| Somatic complaints | 19 (1.3%) | 7 (0.7%) | 26 (1.1%) | 2.03 | 0.155 |
| Schizoid/Compulsive activity | 67 (4.6%) | 32 (3.2%) | 99 (4.1%) | 2.90 | 0.088 |
| Hyperactivity | 53 (3.7%) | 34 (3.4%) | 87 (3.6%) | 0.08 | 0.775 |
| Sexual problem | 26 (1.8%) | 17 (1.7%) | 43 (1.8%) | 0.02 | 0.890 |
| Delinquent behavior | 24 (1.7%) | 17 (1.7%) | 41 (1.7%) | 0.01 | 0.905 |
| Aggressive behavior | 25 (1.7%) | 13 (1.3%) | 38 (1.6%) | 0.65 | 0.422 |
| Cruel | 22 (1.5%) | 11 (1.1%) | 33 (1.4%) | 0.73 | 0.395 |
| Total score | 89 (6.2%) | 43 (4.4%) | 132 (5.4%) | 3.69 | 0.055 |
| **Boys** **of 12-16ys** | **N=2164** | **N=786** | **N=2950** |  |  |
| Somatic complaints | 35 (1.6%) | 13 (1.7%) | 48 (1.6%) | 0.01 | 0.945 |
| Schizoid | 30 (1.4%) | 16 (2.0%) | 46 (1.6%) | 1.58 | 0.208 |
| Social problems | 34 (1.6%) | 19 (2.4%) | 53 (1.8%) | 2.34 | 0.126 |
| Immature | 61 (2.8%) | 13 (1.7%) | 74 (2.5%) | 3.20 | 0.074 |
| Compulsive activity | 80 (3.7%) | 39 (5.0%) | 119 (4.0%) | 2.38 | 0.123 |
| Hostility | 55 (2.5%) | 17 (2.2%) | 72 (2.4%) | 0.35 | 0.556 |
| Delinquent behavior | 34 (1.6%) | 15 (1.9%) | 49 (1.7%) | 0.40 | 0.526 |
| Aggressive behavior | 64 (3.0%) | 18 (2.3%) | 82 (2.8%) | 0.95 | 0.330 |
| Hyperactivity | 118 (5.5%) | 29 (3.7%) | 147 (5.0%) | 3.79 | 0.052 |
| Total score | 217 (10.0%) | 61 (7.8%) | 278 (9.4%) | 3.47 | 0.062 |
| **Girls** **of 12-16ys** | **N=2097** | **N=777** | **N=2874** |  |  |
| Anxiety/Compulsive activity | 51 (2.4%) | 15 (1.9%) | 66 (2.3%) | 0.67 | 0.425 |
| Somatic complaints | 28 (1.3%) | 10 (1.3%) | 38 (1.3%) | 0.01 | 0.920 |
| Schizoid | 140 (6.7%) | 51 (6.6%) | 191 (6.6%) | 0.01 | 0.914 |
| Depression/withdrawal | 55 (2.6%) | 17 (2.2%) | 72 (2.5%) | 0.44 | 0.508 |
| Immature | 51 (2.4%) | 19 (2.4%) | 70 (2.4%) | 0.00 | 0.984 |
| Delinquent behavior | 50 (2.4%) | 15 (1.9%) | 65 (2.3%) | 0.53 | 0.467 |
| Aggressive behavior | 53 (2.5%) | 11 (1.4%) | 64 (2.2%) | 3.22 | 0.073 |
| Cruel | 39 (1.9%) | 18 (2.3%) | 57 (2.0%) | 0.61 | 0.435 |
| Total score | 188 (9.0%) | 65 (8.4%) | 253 (8.8%) | 0.25 | 0.614 |

Abbreviations: COVID-19vs. coronavirus disease 2019; RS group. reopened school group; HS groups. home schooling group

Data are n(%).Chi-square was employed to compare the positive detection rate between the two groups.

**Supplementary Table 2 Demographic and Psychosocial Characteristic of RS Group and HS Group** **During COVID-19**

| **Characteristics** | **RS group**  **(n=7453)** | **HS group**  **(n=3619)** | **Total**  **(n=11072)** | **Effect Size** | **Z/chi-square** | ***P* value** |
| --- | --- | --- | --- | --- | --- | --- |
| Age (years) |  |  |  |  |  |  |
| 6-11 | 9.2(1.32) | 9.0(1.34) | 9.1(1.33) | 0.1721* | -6.00 | <0.001 |
| 12-16 | 13.9(1.38) | 13.9(1.44) | 13.9(1.40) | 0.0259* | -1.02 | 0.31 |
| Sex |  |  |  | 0.0112^#^ | 1.38 | 0.24 |
| Male | 3911 (52.5%) | 1856 (51.3%) | 5767 (52.1%) |  |  |  |
| Female | 3542 (47.5%) | 1763 (48.7%) | 5305 (47.9%) |  |  |  |
| Residential place |  |  |  | 0.0049^#^ | 0.26 | 0.61 |
| Urban | 4260 (57.2%) | 2050 (56.6%) | 6310 (57.0%) |  |  |  |
| Rural | 3193 (42.8%) | 1569 (43.4%) | 4762 (43.0%) |  |  |  |
| Maternal education status |  |  |  | 0.1071^#^ | 126.96 | <0.001 |
| ≤9 years | 4345 (58.3%) | 2511 (69.4%) | 6856 (61.9%) |  |  |  |
| >9years | 3108 (41.7%) | 1108 (30.6%) | 4216 (38.1%) |  |  |  |
| Parents having organic diseases |  |  |  | 0.0395^#^ | 17.31 | <0.001 |
| Yes | 147 (2.0%) | 118 (3.3%) | 265 (2.4%) |  |  |  |
| No | 7306 (98.0%) | 3501 (96.7%) | 10807 (97.6%) |  |  |  |
| Family income |  |  |  | 0.0063^#^ | 0.44 | 0.505 |
| Reduced | 3671 (49.3%) | 1807 (49.9%) | 5478 (49.5%) |  |  |  |
| No change/ Increased | 3782 (50.7%) | 1812 (50.1%) | 5594 (50.5%) |  |  |  |
| Parent-offspring conflict |  |  |  | 0.0431^#^ | 20.58 | <0.001 |
| No | 2391 (32.1%) | 1318 (36.4%) | 3709 (33.5%) |  |  |  |
| Yes | 5062 (67.9%) | 2301 (63.6%) | 7363 (66.5%) |  |  |  |
| Sedentary Time (hours) |  |  |  | 0.0575^#^ | 36.55 | <0.001 |
| ≤6 | 5163 (69.3%) | 2708 (74.8%) | 7871 (71.1%) |  |  |  |
| >6 | 2290 (30.7%) | 911 (25.2%) | 3201 (28.9%) |  |  |  |
| Homework time (hours) |  |  |  | 0.0834^#^ | 76.93 | <0.001 |
| ≤2 | 4114 (55.2%) | 2315 (64.0%) | 6429 (58.1%) |  |  |  |
| >2 | 3339 (44.8%) | 1304 (36.0%) | 4643 (41.9%) |  |  |  |
| Screen exposure time (hours) |  |  |  | 0.0098^#^ | 1.07 | 0.30 |
| ≤4 | 4320 (58.0%) | 2135 (59.0%) | 6455 (58.3%) |  |  |  |
| >4 | 3133 (42.0%) | 1484 (41.0%) | 4617 (41.7%) |  |  |  |
| Physical activity time(hours) |  |  |  | 0.1590^#^ | 279.85 | <0.001 |
| ≤1 | 4338 (58.2%) | 1494 (41.3%) | 5832 (52.7%) |  |  |  |
| >1 | 3115 (41.8%) | 2125 (58.7%) | 5240 (47.3%) |  |  |  |
| Sleep problems |  |  |  | 0.0344^#^ | 13.13 | <0.001 |
| No | 5158 (69.2%) | 2626 (72.6%) | 7784 (70.3%) |  |  |  |
| Yes | 2295 (30.8%) | 993 (27.4%) | 3288 (29.7%) |  |  |  |
| Number of close friends |  |  |  | 0.0090# | 0.89 | 0.344 |
| <4 | 4281 (57.4%) | 2113 (58.4%) | 6394 (57.7%) |  |  |  |
| ≥4 | 3172 (42.6%) | 1506 (41.6%) | 4678 (42.3%) |  |  |  |

Abbreviations: COVID-19vs. coronavirus disease 2019; RS group. reopened school group; HS groups. home schooling group

Data are mean (SD) or n(%). Effect size is estimated by ^*^Cohen’s d or ^#^phi coefficient.

**Supplementary Table 3 Multivariate logistic regression analyses of the presence of at least a positive screened dimension of internalizing and externalizing behavior in different age and sex subgroups of RS group.**

| **Variables** | **β** | **Wald Chi-Square** | **OR (95%CI)** | ***P* value** |
| --- | --- | --- | --- | --- |
| **Boys of 6-11** |  |  |  |  |
| **Internalizing behavior** |  |  |  |  |
| Age (years) | 0.1670 | 5.9256 | 1.182 (1.033 to 1.352) | 0.015 |
| Screen exposure time (>4 hours vs. ≤4 hours per day) | 0.4713 | 6.8806 | 1.602 (1.127 to 2.278) | 0.009 |
| Number of close friends (≥4 vs. <4) | -0.4599 | 6.1547 | 0.631 (0.439 to 0.908) | 0.013 |
| **Externalizing behavior** |  |  |  |  |
| Physical activity (>1 hour vs.≤1 hour per day) | -0.6389 | 7.9428 | 0.528 (0.338 to 0.823) | 0.005 |
| Number of close friends (≥4 vs. <4) | -0.5369 | 5.2811 | 0.585 (0.370 to 0.924) | 0.022 |
| **Girls of 6-11** |  |  |  |  |
| **Internalizing behavior** |  |  |  |  |
| Sedentary time (>6 hours vs.≤6 hours per day) | 0.6979 | 6.5615 | 2.010 (1.178 to 3.428) | 0.010 |
| Number of close friends (≥4 vs. <4) | -0.5720 | 5.3712 | 0.564 (0.348 to 0.916) | 0.020 |
| **Externalizing behavior** |  |  |  |  |
| Residential aera (Urban vs. Rural) | -0.6342 | 6.3706 | 0.530 (0.324 to 0.868) | 0.012 |
| Family income (Reduced vs. No change) | 0.8044 | 9.2997 | 2.235 (1.333 to 3.748) | 0.002 |
| Homework time (>2 hours vs. ≤2 hours per day) | 1.0015 | 14.2174 | 2.722 (1.618 to 4.582) | <.001 |
| Screen exposure time (>4 hours vs. ≤4 hours per day) | 0.6298 | 5.9766 | 1.877 (1.133 to 3.110) | 0.014 |
| Parent-offspring conflict (Yes vs. No) | 1.0398 | 7.8432 | 2.829 (1.366 to 5.856) | 0.005 |
| Number of close friends (≥4 vs. <4) | -0.7543 | 7.3280 | 0.470 (0.272 to 0.812) | 0.007 |
| **Boys of 12-16** |  |  |  |  |
| **Internalizing behavior** |  |  |  |  |
| Age (years) | -0.2501 | 11.6374 | 0.779 (0.675 to 0.899) | <.001 |
| Family income (Reduced vs. No change) | 0.7126 | 12.1258 | 2.039 (1.365 to 3.045) | <.001 |
| Sedentary time (>6 hours vs.≤6 hours per day) | 0.5857 | 9.2346 | 1.796 (1.231 to 2.621) | 0.002 |
| Parents having organic diseases (yes vs. no) | 1.2099 | 9.4323 | 3.353 (1.549 to 7.258) | 0.002 |
| Number of close friends (≥4 vs. <4) | -0.5188 | 6.4121 | 0.595 (0.398 to 0.889) | 0.011 |
| **Externalizing behavior** |  |  |  |  |
| Age (years) |  |  | 0.764 (0.665 to 0.877) | <.001 |
| Family income (Reduced vs. No change) | -0.2696 | 14.5766 | 1.481 (1.025 to 2.140) | 0.037 |
| Sedentary time (>6 hours vs.≤6 hours per day) | 0.3928 | 4.3713 | 1.542 (1.073 to 2.216) | 0.019 |
| Parent-offspring conflict (Yes vs. No) | 0.4334 | 5.4885 | 1.846 (1.152 to 2.957) | 0.011 |
| Parents having organic diseases (yes vs. no) | 0.6128 | 6.4890 | 2.369 (1.015 to 5.530) | 0.046 |
| Number of close friends (≥4 vs. <4) | 0.8626 | 3.9781 | 0.395 (0.261 to 0.598) | <.001 |
| **Girls of 12-16** |  |  |  |  |
| **Internalizing behavior** |  |  |  |  |
| Sedentary time (>6 hours vs.≤6 hours per day) | 0.6417 | 13.1254 | 1.900 (1.343 to 2.688) | <.001 |
| Homework time (>2 hours vs. ≤2 hours per day) | -0.4286 | 5.9115 | 0.651 (0.461 to 0.920) | 0.015 |
| Maternal education status (>9 years vs.≤9years) | -0.5014 | 6.3665 | 0.606 (0.410 to 0.894) | 0.012 |
| Number of close friends (≥4 vs. <4) | -0.5480 | 8.4898 | 0.578 (0.400 to 0.836) | 0.004 |
| **Externalizing behavior** |  |  |  |  |
| Homework time (>2 hours vs. ≤2 hours per day) | -0.9232 | 14.4316 | 0.397 (0.247 to 0.640) | <.001 |
| Screen exposure time (>4 hours vs. ≤4 hours per day) | 0.6951 | 7.3807 | 2.004 (1.214 to 3.309) | 0.007 |
| Parent-offspring conflict (Yes vs. No) | 0.6008 | 4.2454 | 1.824 (1.030 to 3.230) | 0.039 |
| Number of close friends (≥4 vs. <4) | -1.0765 | 12.8723 | 0.341 (0.189 to 0.614) | <.001 |

Abbreviations: OR, odds ratio; CI, confidence interval. Multivariate logistic regression analysis was performed using stepwise variable selection procedure to identify independent predictors contributing to the presence of at least a positive screened dimension of internalizing and externalizing behavior in RS group.

**Supplementary Table 4 Multivariate logistic regression analyses of** **the presence of at least a positive screened dimension of internalizing and externalizing behavior in different age and sex subgroups of HS group.**

| **Variables** | **β** | **Wald Chi-Square** | **OR (95%CI)** | ***P* value** |
| --- | --- | --- | --- | --- |
| **Boys of 6-11** |  |  |  |  |
| **Internalizing behavior** |  |  |  |  |
| Number of close friends (≥4 vs. <4) | -0.6323 | 5.1155 | 0.531 (0.307 to 0.919) | 0.024 |
| Sedentary time (>6 hours vs.≤6 hours per day) | 0.9197 | 12.4704 | 2.509 (1.506 to 4.180) | <.001 |
| **Externalizing behavior** |  |  |  |  |
| Sedentary time (>6 hours vs.≤6 hours per day) | 0.7872 | 5.5797 | 2.197 (1.143 to 4.222) | 0.018 |
| Homework time (>2 hours vs. ≤2 hours per day) | 0.8907 | 7.9167 | 2.437 (1.310 to 4.532) | 0.005 |
| Number of close friends (≥4 vs. <4) | -1.3140 | 8.6983 | 0.269 (0.112 to 0.644) | 0.003 |
| **Girls of 6-11** |  |  |  |  |
| **Internalizing behavior** |  |  |  |  |
| Homework time (>2 hours vs. ≤2 hours per day) | 0.7865 | 5.7047 | 2.196 (1.152 to 4.187) | 0.017 |
| Screen exposure time (>4 hours vs. ≤4 hours per day) | 0.6761 | 4.1636 | 1.966 (1.027 to 3.764) | 0.041 |
| Physical activity (>1 hour vs.≤1 hour per day) | -0.9941 | 9.1641 | 0.370 (0.194 to 0.704) | 0.002 |
| Parents having organic diseases (yes vs. no) | 1.7844 | 10.6386 | 5.956 (2.038 to 17.403) | 0.001 |
| **Externalizing behavior** |  |  |  |  |
| Family income (Reduced vs. No change) | 0.9923 | 7.5972 | 2.697 (1.332 to 5.462) | 0.006 |
| Homework time (>2 hours vs. ≤2 hours per day) | 1.0101 | 9.4631 | 2.746 (1.443 to 5.226) | 0.002 |
| Number of close friends (≥4 vs. <4) | -1.8357 | 11.6321 | 0.160 (0.056 to 0.458) | <.001 |
| Sedentary time (>6 hours vs.≤6 hours per day) | 0.9968 | 7.7976 | 2.710 (1.346 to 5.455) | 0.005 |
| Physical activity (>1 hour vs.≤1 hour per day) | -0.6940 | 4.5093 | 0.500 (0.263 to 0.948) | 0.034 |
| Parents having organic diseases (yes vs. no) | 1.5254 | 6.8132 | 4.597 (1.462 to 14.451) | 0.009 |
| **Boys of 12-16** |  |  |  |  |
| **Internalizing behavior** |  |  |  |  |
| Homework time (>2 hours vs. ≤2 hours per day) | -0.8023 | 5.0610 | 0.448 (0.223 to 0.902) | 0.024 |
| Physical activity (>1 hour vs.≤1 hour per day) | -0.6650 | 4.4561 | 0.514 (0.277 to 0.954) | 0.035 |
| **Externalizing behavior** |  |  |  |  |
| Homework time (>2 hours vs. ≤2 hours per day) | -0.9023 | 5.1114 | 0.406 (0.186 to 0.887) | 0.024 |
| Parent-offspring conflict (Yes vs. No) | 0.8811 | 4.0358 | 2.414 (1.022 to 5.702) | 0.045 |
| Parents having organic diseases (yes vs. no) | -0.9733 | 6.6188 | 3.689 (1.003 to 13.564) | 0.049 |
| Physical activity (>1 hour vs.≤1 hour per day) | 1.3053 | 3.8602 | 0.378 (0.180 to 0.793) | 0.010 |
| **Girls of 12-16** |  |  |  |  |
| **Internalizing behavior** |  |  |  |  |
| Sedentary time (>6 hours vs.≤6 hours per day) | 0.6579 | 5.6681 | 1.931 (1.123 to 3.318) | 0.017 |
| **Externalizing behavior** |  |  |  |  |
| Physical activity (>1 hour vs.≤1 hour per day) | -0.8602 | 3.9122 | 0.423 (0.180 to 0.992) | 0.048 |

Abbreviations: OR, odds ratio; CI, confidence interval. Multivariate logistic regression analysis was performed using stepwise variable selection procedure to identify independent predictors contributing to the presence of at least a positive screened dimension of internalizing and externalizing behavior in HS group
